# Human MRSA Isolates with Novel Genetic Homolog, Germany

**DOI:** 10.3201/eid1806.110910

**Published:** 2012-06

**Authors:** André Kriegeskorte, Britta Ballhausen, Evgeny A. Idelevich, Robin Köck, Alexander W. Friedrich, Helge Karch, Georg Peters, Karsten Becker

**Affiliations:** University Hospital Münster, Germany (A. Kriegeskorte, B. Ballhausen, E.A. Idelevich, R. Köck, H. Karch, G. Peters, K. Becker);; University Medical Center Groningen, Groningen, the Netherlands (A.W. Friedrich)

**Keywords:** Staphylococcus aureus, methicillin-resistant Staphylococcus aureus, MRSA, mecA_LGA251_, mecA, antibiotic, antibacterial, antimicrobial, drug resistance, bacteria, MIC, Germany, genetics, homolog

**To the Editor:** Methicillin-resistant *Staphylococcus aureus* (MRSA) represents a major cause of hospital-, community- and livestock-acquired infections that are increasingly difficult to manage (*1**–**3*). Detection and identification of MRSA by culture and nucleic acid–based methods is challenged by heterogeneous penicillin-binding protein 2a (PBP2a) expression and variability of the staphylococcal cassette chromosome (SCC*mec*) elements. Recently, a new SCC*mec* element (XI) carried in bovine and human isolates was described (*4**,**5*). This SCC*mec* element contains a novel *mecA* homolog, designated *mecA*_LGA251_, that is not detectable by usual *mecA*-specific PCR approaches and PBP2a agglutination tests. García-Álvarez et al. reported this novel *mecA* homolog exhibited 70% identity at DNA level to the *mecA* gene, and suggested these strains were transmitted from livestock to humans (*4*).

To search for isolates possessing the novel *mecA*_LAG251_, we screened *S. aureus* databases for those entries describing oxacillin/cefoxitin-resistant phenotypes that were negative for *mecA* by PCR (*6*) or harbored *S. aureus* protein A gene (*spa*) types known to be associated with the occurrence of *mecA*_LGA251_ (*4**,**5*). The databases of the University Hospital Münster contain *S. aureus spa* typing results of *S. aureus* isolates obtained from hospital admission screenings and specimens from patients treated at University Hospital Münster. Moreover, they include isolates derived from human and animal subjects, respectively, of 2 cross-border projects between the Netherlands and Germany: MRSA-net EUREGIO Twente/Münsterland and SafeGuard MRSA vet-net (*2**,**7*).

The presence of *mecA*_LGA251_ was verified by using a specific PCR that applied newly designed primers: mecAL1 (5′-AGC TGG CCA TCC CTT TAT TT-3′) and mecAL2 (5′-CTG GCA TAT GGA GAA GAA GAA A-3′), derived from the sequence of *S. aureus* LGA251 provided by M. Holden (Wellcome Trust Sanger Institute, Hinxton, UK; accession no. FR821779). The sensitivities and specificities of primers were checked by applying *S. aureus* and other staphylococcal isolates of different clonal backgrounds (*8**,**9*). Positive PCR products were sequenced to confirm identification of *mecA*_LGA251_; the isolates were then characterized by typing the SCC*mec* region with specific primers for *mecR1*, *mecI*, *blaZ*, *ccrA*, and *ccrB* related to type XI SCC*mec* as described by García-Álvarez et al. (*4*). Identified isolates were tested for PBP2a by using a latex agglutination assay (Oxoid Deutschland GmbH, Wesel, Germany). We used Etest (bioMérieux SA, Marcy-l'Étoile, France) for antibacterial agent susceptibility testing of β-lactams and other antibacterial agents.

We report on 16 (clinically derived, n = 14; ovine origin, n = 2) oxacillin/cefoxitin-resistant *S. aureus* isolates possessing the recently described *mecA*_LGA251_ isolate, but lacking the classical *mecA* gene currently defining classic MRSA (Table). The isolates belong to *spa* types t843, t978, t1535, t1773, and t7189. Concurring with the findings in the United Kingdom and Denmark, we found t843 to be the most prevalent *spa* type. Results of the PBP2a latex agglutination assay were negative for all isolates except for 1 (no. 14), which was indeterminable. García-Álvarez et al. described negative results for all tested isolates (*4*); Shore et al. reported inconsistent results with this test (*5*).

**Table:** Description of *mecA*_LGA251_-positive isolates regarding their *spa* type, ability to grow on selective MRSA media, PBP2a agglutination, *mec* gene possession, and SCC*mec*-type*

Isolate no. and origin	Characteristics							
	Year of isolation	Specimen	*spa* type	Growth on selective MRSA medium†	PBP2a agglutination	Presence of		
						*mecA*	*mecA*_LGA251_	SCC*mec*XI
Human								
1	2010	Nasal swab	t843	+	–	–	+	+
2	2010	Wound	t843	+	–	–	+	+
3	2010	Wound	t843	+	–	–	+	+
4	2010	Nasal swab	t843	+	–	–	+	+
5	2011	Nasal swab	t843	+	–	–	+	+
6	2004	Sputum	t843	+	–	–	+	+
7	2010	Nasal swab	t843	+	–	–	+	+
8	2007	Mouth swab	t843	+	–	–	+	+
9	2010	Nasal swab	t843	+	–	–	+	+
10	2011	Nasal swab	t843	+	–	–	+	+
11	2011	Joint aspirate	t843	+	–	–	+	+
12	2007	Nasal swab	t978	+	–	–	+	+
13	2010	Nasal swab	t7189	+	–	–	+	+
14	2009	Nasal swab	t1773	+	ND	–	+	+
Sheep						–		
15	2010	Unknown	t1535	+	–	–	+	+
16	2010	Unknown	t1535	+	–	–	+	+

*spa, *Staphylococcus aureus* protein A; MRSA, methicillin-resistant *S. aureus*; PBP2a, penicillin-binding protein 2a; SCC, staphylococcal cassette chromosome; +, positive; –, negative; ND, not done. †ChromID MRSA-Plates (bioMérieux, Marcy-l'Étoile, France).

According to the Clinical and Laboratory Standards Institute MIC interpretative standards for staphylococci (*10*), antibacterial agent susceptibility testing revealed resistance to benzylpenicillin and oxacillin/cefoxitin for all isolates. All isolates were shown to produce β-lactamases. Apart from the general categorization of oxacillin/cefoxitin-resistant isolates as resistant to all β-lactams, the MICs of drugs for all isolates included were read as susceptible for imipenem (MIC for 90% of strains tested 0.5 µg/mL) as well as for the anti-MRSA cephalosporin ceftobiprole (MIC for 90% of strains tested 1 µg/mL applying provisional breakpoint <4 µg/mL). A large range of MICs were observed for classic cephalosporins, ranging from those isolates categorized as susceptible (cephalothin, n = 15; cefuroxime, n = 10; ceftriaxone, n = 2; cefepime, n = 9) to those classified as resistant.

We observed relatively low oxacillin/cefoxitin MICs for some of the *mecA_LAG251_*-positive isolates (MIC 3 µg/mL, n = 1; MIC 4 µg/mL, n = 1; MIC 8 µg/mL, n = 3) compared with the MRSA reference strain ATCC 43300 (MIC 32 µg/mL). All isolates tested were susceptible to all non–β-lactam antibacterial agents, comprising glycopeptides, lipopeptides, fluoroquinolones, macrolides, lincosamides, oxazolidinones, rifampins, streptogramins, glycylcyclines, folate pathway inhibitors, aminoglycosides, and fosfomycin.

Until *mecA*_LGA251_ is included as a diagnostic target in molecular MRSA detection tests, oxacillin/cefoxitin-resistant isolates determined to be methicillin-susceptible by traditional, culture-based susceptibility testing methods should not be disregarded, even if *mecA* and/or PBP2a tests fail to detect their targets. Susceptibility patterns of *mecA*_LGA251_-positive *S. aureus* isolates revealed low MICs of oxacillin compared with those for MRSA of the classical *mecA* type. We presume this indicates an altered affinity of β-lactam antibacterial agents to the putative *mecA*_LGA251_ gene product or a divergent expression of the gene. The choice and the dosage of antibacterial agents applicable for *S. aureus* infections should be reconsidered in light of this novel *mecA* homolog in molecular screening and identification tests. Studies are warranted to investigate the prevalence of this novel MRSA entity in and outside of hospitals in the human population and in livestock, its clinical effects, and its response to antibacterial agent therapy.
